# B7-CD28 gene family expression is associated with prognostic and immunological characteristics of diffuse large B-cell lymphoma

**DOI:** 10.18632/aging.102025

**Published:** 2019-06-13

**Authors:** Gangjian Wang, Xiaorui Fu, Yu Chang, Xin Li, Xiaolong Wu, Ling Li, Lei Zhang, Zhenchang Sun, Xudong Zhang, Mingzhi Zhang

**Affiliations:** 1 Department of Oncology, The First Affiliated Hospital of Zhengzhou University, Zhengzhou, China

**Keywords:** diffuse large B-cell lymphoma, risk score, prognosis, immune target

## Abstract

The B7-CD28 gene family plays a key role in regulating cellular immunity and is closely related to tumorigenesis and immune evasion. Here, we explored associations between clinical and immune features and B7-CD28 gene family expression in Gene Expression Omnibus (GEO) datasets representing 1812 diffuse large B-cell lymphoma (DLBCL) patients. This included 414 in the GSE10846 training cohort and 470 and 928 patients in the GSE31312 and GSE117556 validation cohorts, respectively. Four survival-associated genes identified in the GSE10846 cohort by univariate Cox analysis were incorporated into a multivariate analysis, ultimately establishing a three-gene risk signature. Risk scores assigned based on expression of these genes were validated by Kaplan–Meier and multivariable Cox analyses in the remaining datasets and in important clinical subsets. High-risk patients had shorter overall survival and, in some cases, progression-free survival than low-risk patients. Additionally, expression of programmed cell death 1 (*PD-1*) and programmed death ligand 1 (*PD-L1*), as well as several other important immune checkpoint genes, differed between high-risk and low-risk patients, as did the proportions of various immune-infiltrating cells. Finally, further analysis confirmed that these B7-CD28 genes play important roles in immune responses altered in DLBCL.

## INTRODUCTION

Diffuse large B-cell lymphoma (DLBCL) is the most common and aggressive form of non-Hodgkin's lymphoma, accounting for 30–35% of all non-Hodgkin's lymphomas in most western countries [1]. Unfortunately, 30–40% of patients experience relapse and develop refractory DLBCL that is resistant to most chemotherapy regimens [2]. Programmed cell death 1/programmed death ligand 1 (PD-1/PD-L1) and CTLA-4 are the most common targets of immune-blocking cancer treatments, and many clinical trials are currently investigating the use of corresponding monoclonal antibodies. However, despite a short initial period of positive response, most patients receiving these treatments soon experience disease progression [3, 4]. Treatments that combine various immune checkpoint inhibitors and agonists may be more effective. For example, combined anti-PD-1 and anti-CTLA-4 treatment results in synergistic effects and increased antitumor activity (NCT03305445).

Immune evasion is a hallmark of malignant tumors and represents an important step in tumor formation [5], and the B7-CD28 gene family plays an important role in immune evasion by tumors [6]. PD-1 and CTLA-4 are members of the CD28 family, and PD-L1 is a member of the B7 family. B7 family ligands and CD28 family receptors are essential for immune responses and proper T cell function. B7 ligands are widely expressed in antigen-presenting cells (APCs), while CD28 receptors are widely expressed on T cells; interactions between these ligands and receptors can stimulate or inhibit T cell activation [7, 8]. Therefore, in addition to PD-1/PD-L1 and CTLA-4, other B7-CD28 family members may be targets for antitumor immunotherapy [9, 10]. Due to the limitations of monomolecular targeted therapies, combined multi-molecular therapies and overall B7-CD28 expression patterns warrant further investigation.

In this study, we 1) systematically explored the prognostic value of the B7-CD28 family in DLBCL, 2) established a prognostic model based on B7-CD28 expression, and 3) further characterized its clinical characteristics and significance in two large patient cohorts. We then used the CIBERSORT algorithm to evaluate 22 immune infiltrating cell types, examine B7-CD28 expression in these cells, and examine the relationship between B7-CD28 expression and clinically important immune checkpoints. Finally, we investigated the relationships between the B7-CD28 family and immune responses and T cell-based immunity. This is the first and most comprehensive study of associations between B7-CD28 family genes and their clinical, molecular, and immunological characteristics in DLBCL. Our findings may help to optimize immunotherapies for these patients.

## RESULTS

### Three B7-CD28 family genes predict overall survival in DLBCL

**Figure 1:**
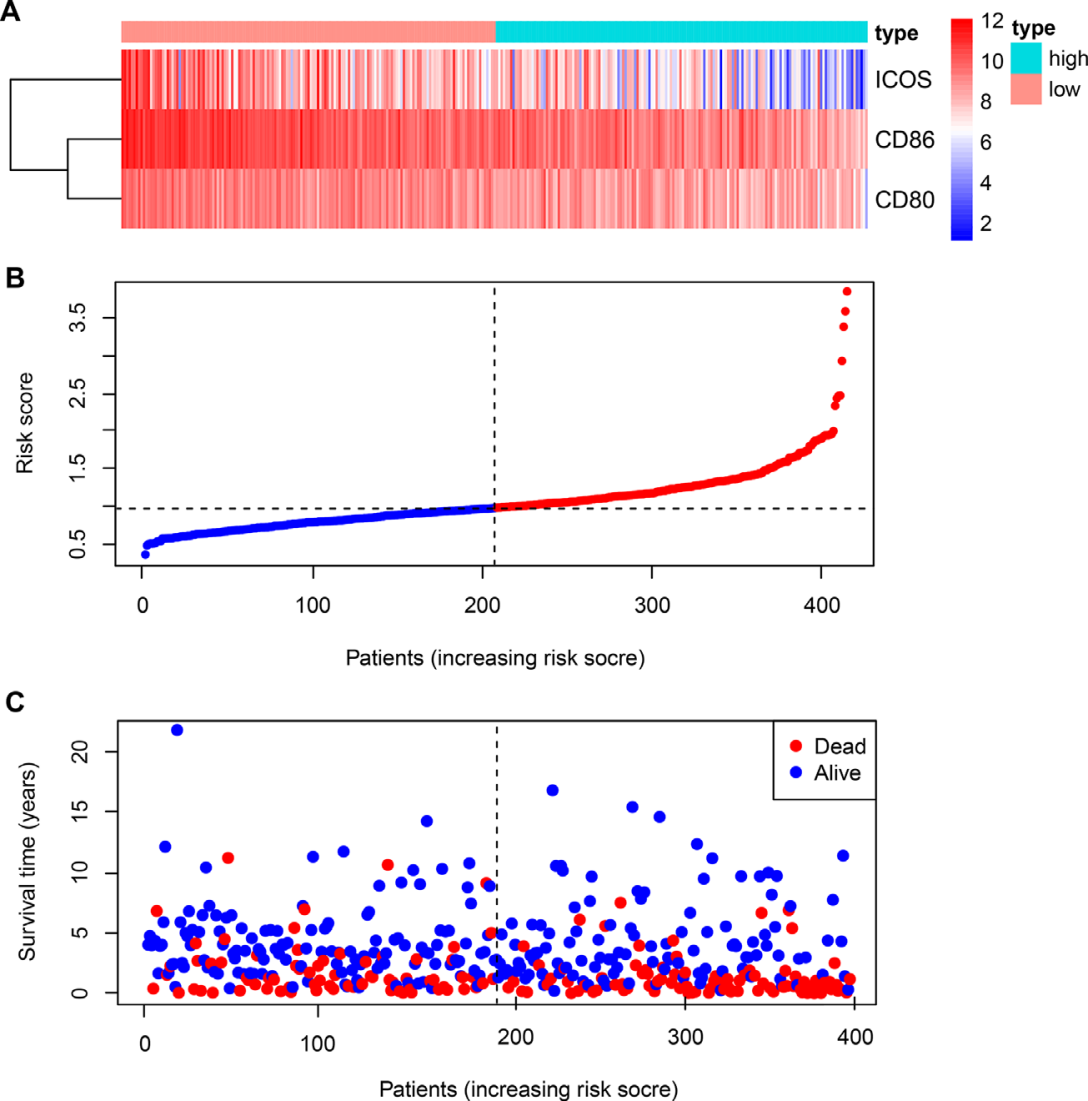
**Expression of B7-CD28 family genes and risk score distribution in GSE10846 patients.** (**A**) Expression distribution of B7-CD28 family genes. (**B**) Gene expression scores for all GSE10846 patients plotted in ascending order of risk score. (**C**) Follow-up and survival of each patient.

**Figure 2:**
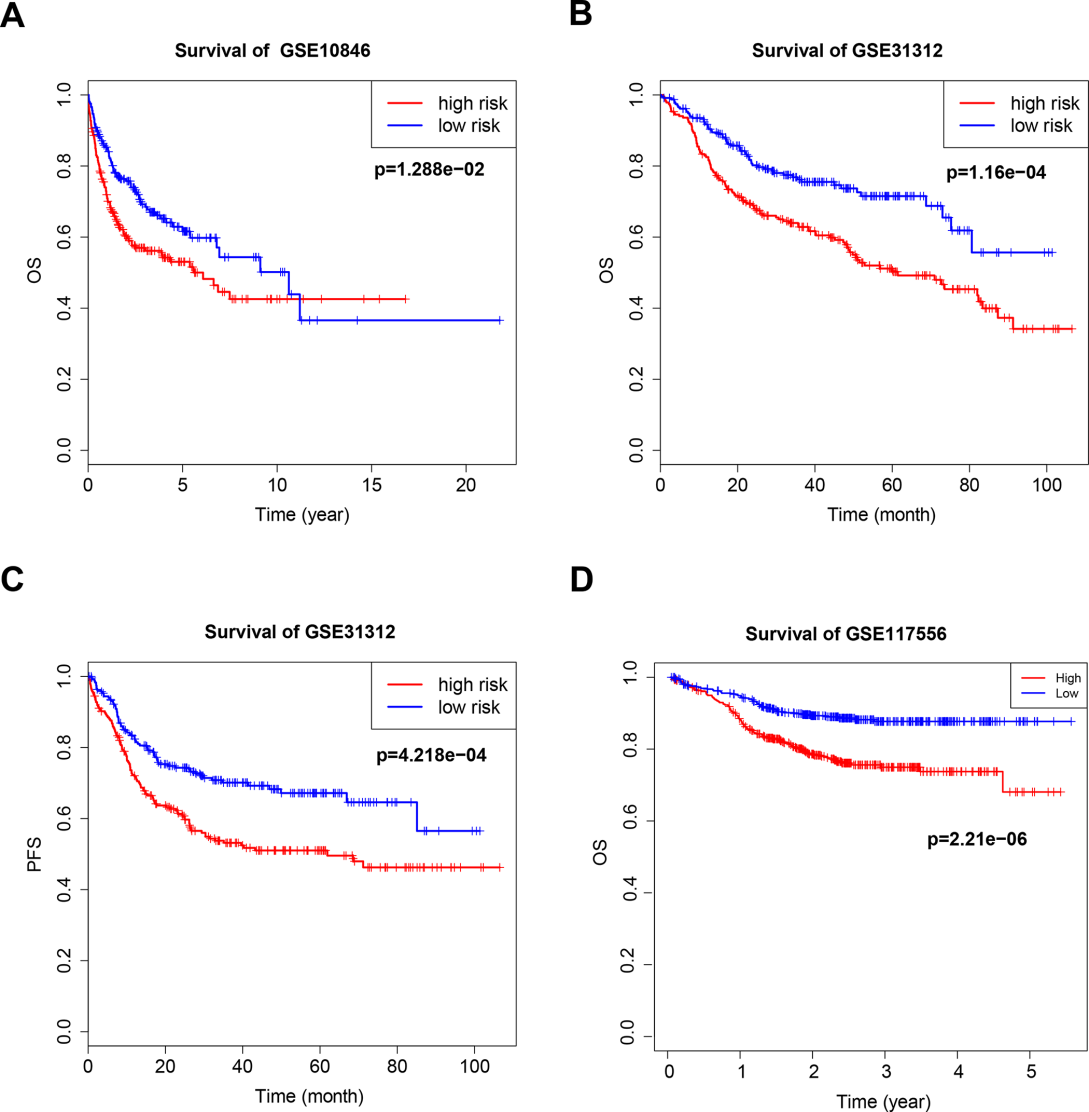
**Kaplan–Meier curves of overall survival (OS) and progression-free survival (PFS) for high-risk and low-risk GSE10846** (**A**), GSE31312 (**B**, **C**) and GSE117556 (**D**) patients.

**Figure 3:**
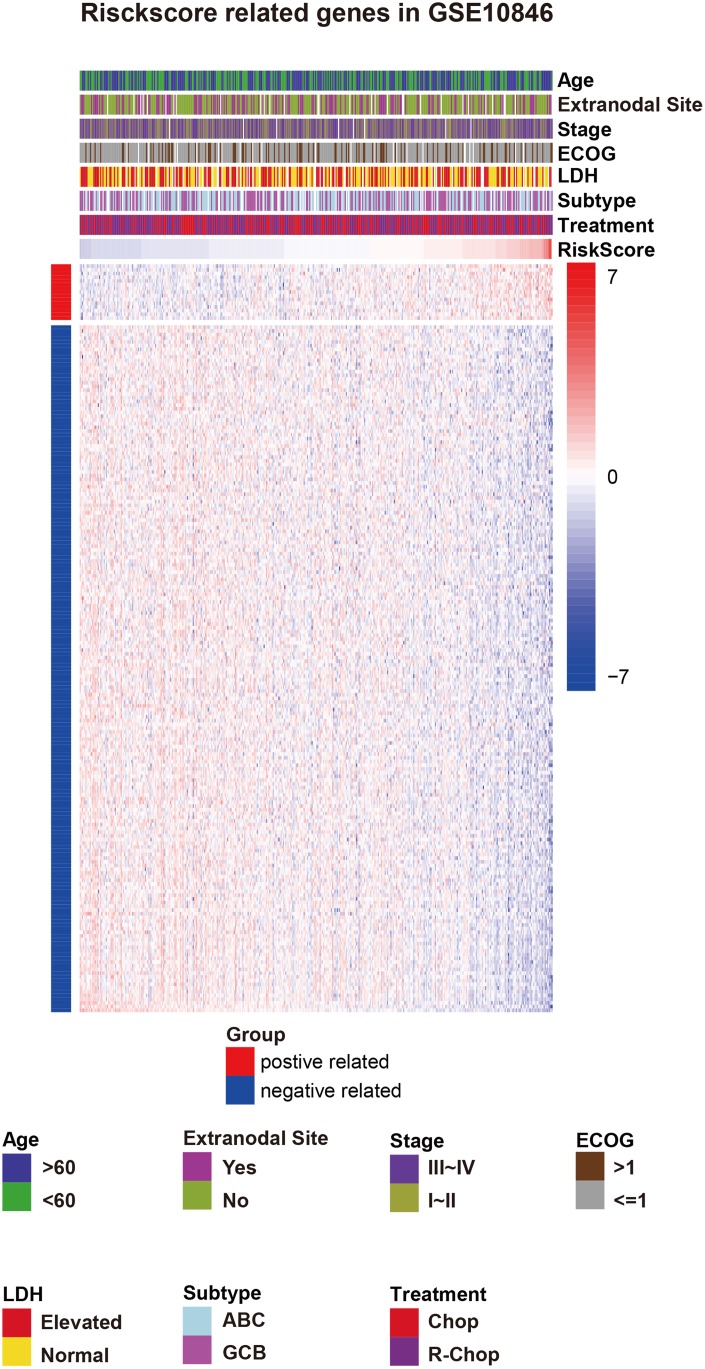
**Risk score-related genes and clinical characteristics in GSE10846 patients.** Abbreviations: ECOG: Eastern Cooperative Oncology Group score, LDH: lactate dehydrogenase, IPI: International Prognostic Index, ABC: activated B-cell-like, GCB: germinal center B-cell-like, CHOP: cyclophosphamide, doxorubicin, vincristine, and prednisone, R-CHOP: cyclophosphamide, doxorubicin, vincristine, and prednisone plus rituximab.

Data from 414 DLBCL patients in the GSE10846 [11] dataset were used as a training cohort, and data from 471 DLBCL patients in the GSE31312 [12] dataset and 928 DLBCL patients in the GSE117556 [13] dataset were used as validation cohorts; their clinical characteristics are summarized in Table 1. The expression of fifteen well-defined B7-CD28 genes in the training cohort was analyzed by univariate Cox regression; of these, *CD86, ICOS, CD80,* and *CTLA4* expression were significantly associated with overall survival (OS) (*P* < 0.05, Table 2). Specifically, increased expression of these four genes was associated with longer survival time. These genes were incorporated into a multivariable Cox proportional hazards regression model using backward conditional stepwise regression; ultimately, a three-gene prognostic model was established using *CD86, ICOS*, and *CD80*. The risk score was calculated as follows: risk score = -*0.2294 × CD86 - 0.2152 × CD80 - 0.0607× ICOS*. Risk scores were calculated for each patient, and the median risk score was used as a cutoff to divide all patients into high-risk and low-risk groups (Figure 1). OS was shorter for patients in the high-risk group compared to those in the low-risk group (*P* = 0.01288) (Figure 2A). However, there were no significant correlations between clinical variables and risk scores (Figure 3, Supplementary Figure 1). Finally, risk score remained an independent prognostic factor (hazard ratio [HR]: 1.714, 95% confidence interval [CI]: 1.080–2.720, *P* = 0.022) even after it was incorporated into a multivariate Cox proportional hazards regression model together with important clinical variables (Table 3).

**Table 1:** Clinical characteristics of the patients in GSE10846 and GSE31312.

**Characteristics**	**GSE10846 (n=414)(%)**	**GSE31312 (n=470) (%)**	**GSE117556 (n=928) (%)**
Age, No.(%)			
<=60	188(45.4)	200(42.6)	332(35.8)
>60	226(54.6)	270(57.4)	596(64.2)
Subtype, No.(%)			
GCB	183(44.2)	227(48.3)	475(51.2)
ABC	167(40.3)	199(42.3)	274(26.3)
Na	64(15.5)	44(9.4)	209(22.5)
Stage, No.(%)			
I~II	189(45.7)	220(46.8)	268(30.8)
III~IV	217(52.4)	229(48.7)	638(68.8)
Na	8(1.9)	21(4.5)	4(0.4)
ECOG, No.(%)			
<=1	296(71.5)	374(79.6)	823(88.7)
>1	93(22.5)	96(20.4)	105(11.3)
NA	25(6)		
LDH			
Normal	173(41.8)	148(31.5)	
Elevated	178(43.0)	278(59.1)	
Na	63(15.2)	44(9.4)	
Extranodal site, No.(%)			
No	238(57.5)	194(41.3)	407(43.9)
Yes	145(35)	276(58.7)	521(56.1)
Na	31(7.5)		
Treatment, No.(%)			
R-Chop	233(56.3)		469(50.5)
Chop	181(43.7)		
RB-Chop			459(49.5)
IPI,			
<=2		274(58.3)	482(51.9)
>2		150(31.9)	446(48.1)
Na		46(9.8)	
Tumor size, N0.(%)			
<=5		268(57.0)	354(381)
>5		98(20.9)	574(61.9)
Na		104(22.1)	
B sympyom, No(%)			
No		276(58.7)	
Yes		132(28.1)	
Na		62(13.2)	

Abbreviations: Na: not available; ECOG: Eastern Cooperative Oncology Group score; LDH: Lactate dehydrogenase; IPI: international prognostic index.

**Table 2:** B7-CD28 gene univariate Cox proportional hazards models in GSE10846.

**Official symbol**	**Aliases**	**Hazard ratio**	**p-value**	**Family**
HHLA2	B7-H5, B7-H7	1.046	0.468	B7 Family
BTLA	CD272	1.127	0.074	CD28 Family
CD28	Tp44	0.910	0.121	CD28 Family
CD86	B7-2, CD28LG2	0.725	0.001	B7 Family
CD80	B7, CD28LG1	0.772	0.007	B7 Family
ICOS	AILIM, CD278	0.904	0.003	CD28 Family
NCR3	NKp30, CD337	0.956	0.349	CD28 Family
VSIR	B7-H5, C10orf54	0.903	0.318	B7 Family
ICOSLG	B7-H2, CD275	0.944	0.307	B7 Family
PDCD1	PD1, CD279	0.980	0.743	CD28 Family
PDCD1LG2	PD-L2, CD273	0.926	0.127	B7 Family
CD274	B7-H1, PD-L1	1.142	0.102	B7 Family
CD276	B7-H3	1.011	0.892	B7 Family
CTLA4	CD152	0.836	0.025	CD28 Family
VTCN1	B7-H4	1.070	0.212	B7 Family

**Table 3:** Multivariate Cox proportional hazards regression analysis of the B7-CD28 signature and clinical characteristics in GSE10846 and GSE 31312.

**Variable**	**GSE10846 OS**			**GSE31312 OS**			**GSE31312 PFS**			**GSE117556 OS**		
	**HR**	**95%CI**	**p-value**	**HR**	**95%CI**	**p-value**	**HR**	**95%CI**	**p-value**	**HR**	**95%CI**	**p-value**
Riskscore	1.714	1.08~2.72	0.022	1.090	1.026~1.158	0.005	1.62	1.106~2.372	0.013	5.47	1.98~15.117	0.001
Age	2.267	1.502~3.42	<0.001	1.696	1.07~2.689	0.025	1.107	0.728~1.683	0.634	1.374	0.87~2.169	0.173
Subtype	2.238	1.506~3.328	<0.001	1.434	0.95~2.165	0.086	1.378	0.925~2.052	0.115	1.47	1.01~2.139	0.44
Stage	1.170	0.789~1.734	0.436	1.879	1.083~3.259	0.025	2.017	1.192~3.414	0.009	1.216	0.134~-2.016	0.447
ECOG	1.826	1.202~2.773	0.005	1.515	0.903~2.54	0.115	1.323	0.804~2.176	0.271	2.021	1.267~3.224	0.003
LDH	1.970	1.312~2.958	0.001	1.102	0.674~1.802	0.699	1.17	0.721~1.899	0.526			
Extranodal site	1.339	0.842~2.127	0.217	1.139	0.727~1.783	0.570	1.078	0.709~1.641	0.724	0.795	0.536~1.180	0.255
Treatment	1.978	1.251~3.127	0.004							0.864	0.598~1.25	0.439
IPI				1.812	1.024~3.207	0.041	1.737	0.995~3.034	0.052	2.117	1.291~3.471	0.003
Tumor size				1.402	0.908~2.164	0.127	1.01	0.652~1.564	0.965	1.230	0.809~1.869	0.333
B symptoms				0.978	0.641~1.493	0.919	1.023	0.684~1.529	0.912			

Abbreviations: OS: overall survival; PFS: progression free survival; ECOG: Eastern Cooperative Oncology Group score; LDH: Lactate dehydrogenase; IPI: international prognostic index; HR: Hazard Ratio; 95%CI: 95% confidence interval

### B7-CD28 three-gene signature is associated with outcomes in DLBCL patients

The B7-CD28 three-gene risk score was significantly associated with OS (*P* < 0.001, Figure 2B, 2D) and progression-free survival (PFS; *P* < 0.001, Figure 2C) in validation cohort patients. As in the training cohort, there were no significant correlations between clinical variables and risk scores in validation cohort patients. Finally, risk score was an independent prognostic factor for both OS and PFS when incorporated into a multivariate Cox proportional hazards regression model together with important clinical variables (Table 3).

### Validation of prognostic signature in important clinical subgroups

**Figure 4:**
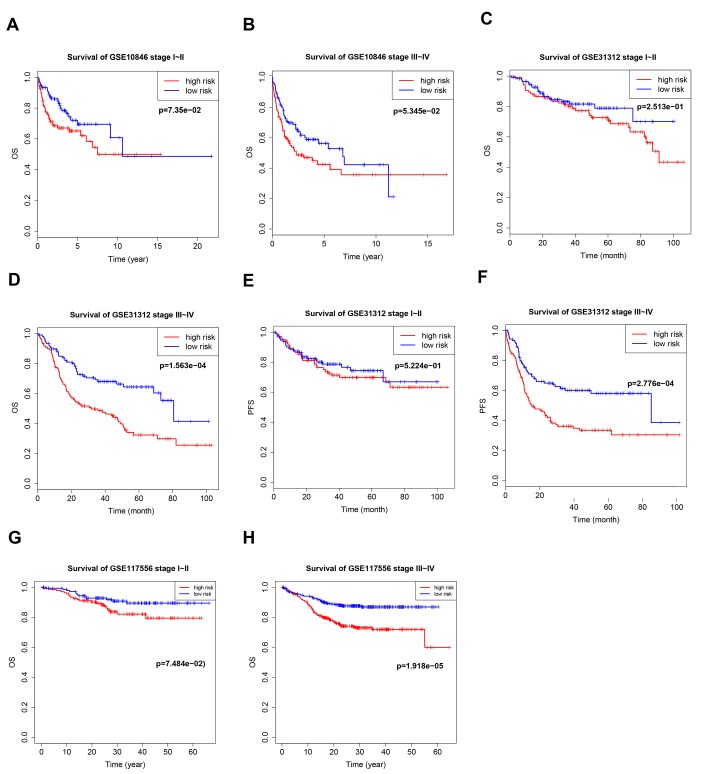
**Kaplan–Meier curves of overall survival (OS) and progression-free survival (PFS) in high-risk and low-risk GSE10846** (**A**, **B**), GSE31312 (**C**–**F**), and GSE117556 (**G**, **H**) patients with different disease stages.

Patients with stage I–II cancer have a very different prognosis than patients with stage III–IV cancer, and the efficacy of different treatment methods and cycles depends on stage. We therefore evaluated B7-CD28 gene expression patterns at different cancer stages separately in high-risk and low-risk patients. Our model accurately predicted OS and PFS in stage III–IV patients, but its predictive accuracy was relatively poor in stage I–II patients (Figure 4).

**Figure 5:**
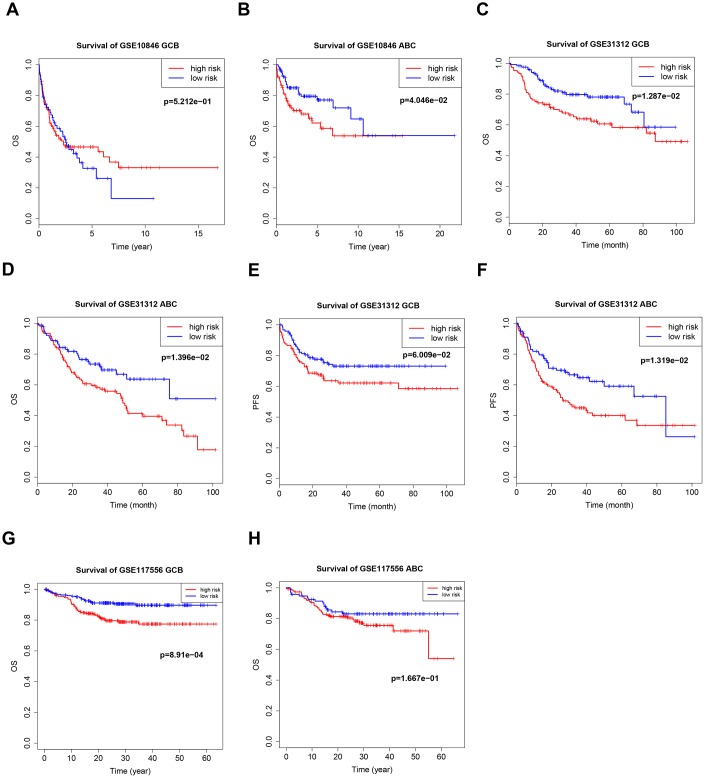
**Kaplan–Meier curves of overall survival (OS) and progression-free survival (PFS) in high-risk and low-risk GSE10846** (**A**, **B**), GSE31312 (**C**–**F**), and GSE117556 (**G**, **H**) patients with different cancer subtypes.

**Figure 6:**
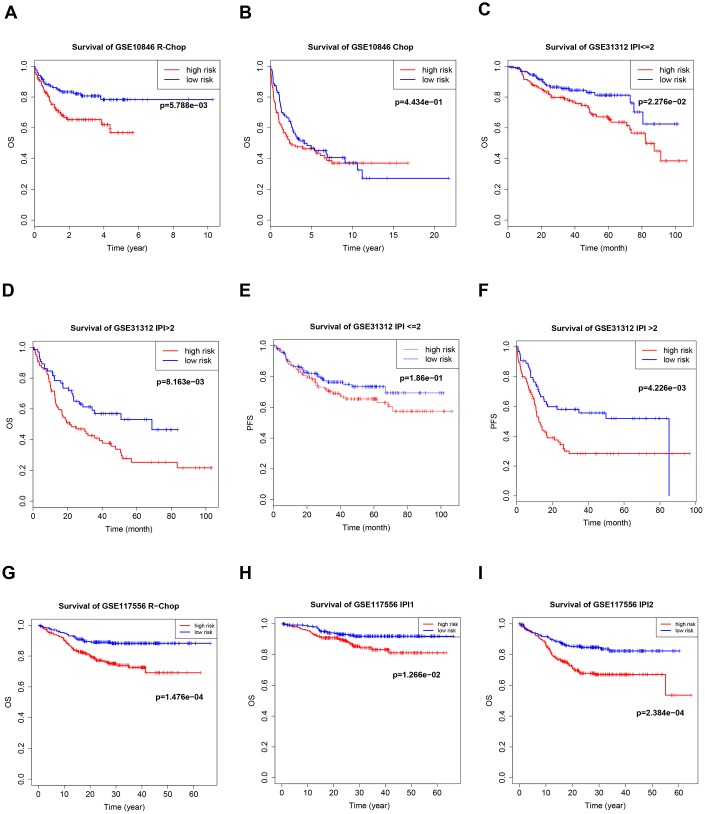
**Kaplan–Meier curves of overall survival (OS) in high-risk and low-risk patients treated with cyclophosphamide, doxorubicin, vincristine, and prednisone (CHOP)** (**B**) or CHOP plus rituximab (R-CHOP) (**A**, **G**). Kaplan–Meier curves of overall survival (OS) and PFS in high-risk and low-risk patients with different international prognostic index (IPI) values (**C**–**F**, **H**, **I**).

Germinal center B-cell-like (GCB) and activated B-cell-like (ABC) are two important subtypes of DLBCL; different treatment regimens are typically used for each subtype, and they have different prognoses. There was a distinct B7-CD28 three-gene risk score cutoff between high-risk and low-risk groups regardless of DLBCL subtype (Figure 5). Because rituximab treatment is cost-prohibitive for many patients, we performed a subgroup analysis of different treatments (cyclophosphamide, doxorubicin, vincristine, and prednisone plus rituximab (R-CHOP) or cyclophosphamide, doxorubicin, vincristine, and prednisone (CHOP)); OS was significantly shorter in high-risk patients in the R-CHOP, but not the CHOP, subgroup (Figure 6). We also validated our model in subgroups established according to the International Prognostic Index (IPI), an important prognostic indicator used for all types of lymphomas; OS was significantly shorter only in high-risk patients with IPI > 2 (Figure 6).

### Correlation between B7-CD28 three-gene signature and immune cell infiltration

**Figure 7:**
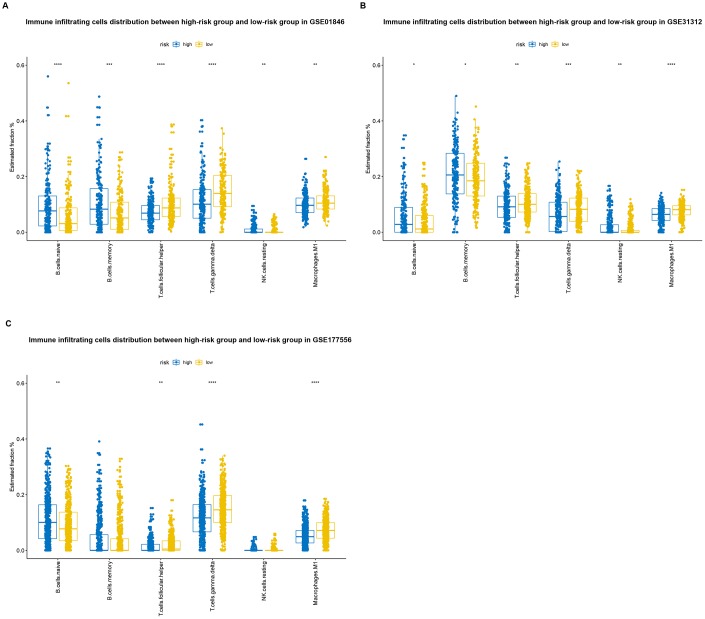
**Distributions of immune-infiltrating cells in high-risk and low-risk GSE10846** (**A**), GSE31312 (**B**), and GSE117556 (**C**) patients.

CIBERSORT and the LM22 signature matrix were used together to estimate proportions of twenty-two immune cell types in samples from each training cohort patient [14], and to evaluate differences in the proportions of each immune cell type between the high-risk and low-risk groups. High-risk patients had significantly higher proportions of memory B cells, naive B cells, and resting (natural killer) NK cells, and significantly lower proportions of follicular helper T cells, gamma delta T cells, and M1 macrophages, than low-risk patients (Figure 7A). Similar results were obtained in the GSE31312 validation cohort (Figure 7B), and the observed effects were even stronger in the GSE117556 validation cohort (Figure 7C).

### Correlation between B7-CD28 expression patterns and immune checkpoints

**Figure 8:**
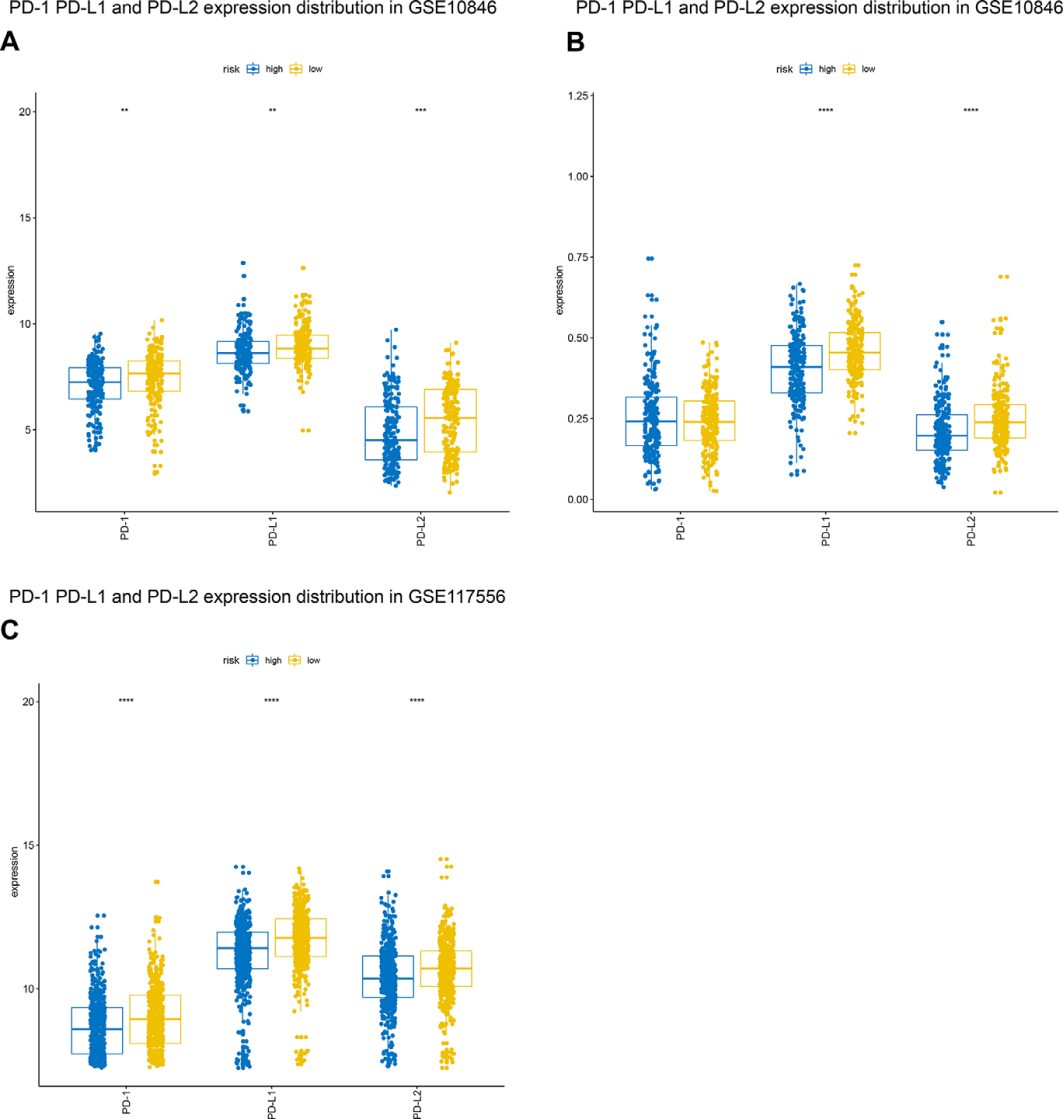
**Distribution of programmed cell death 1 (PD-1), programmed death ligand 1 (PD-L1), and programmed death ligand 2 (PD-L2) expression in GSE10846** (**A**), GSE31312 (**B**), and GSE117556 (**C**) patients.

**Figure 9:**
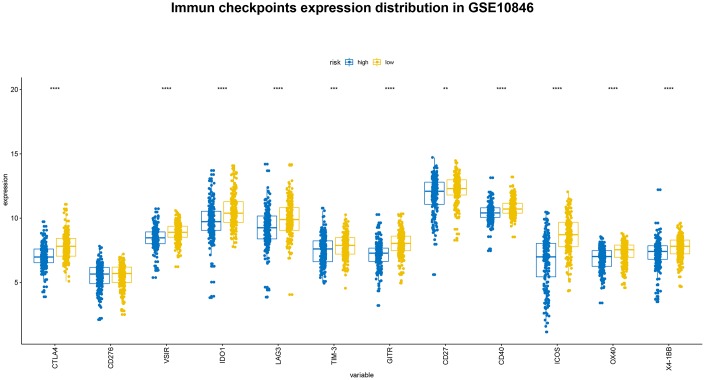
Distribution of immune checkpoint gene expression in GSE10846 patients.

For some tumors, immunotherapy targeting PD-1 and PD-L1 is more effective in patients with high expression of these genes than in patients with low expression [15, 16]. We evaluated PD-1, PD-L1, and PD-L2 expression in the high-risk and low-risk groups. Both PD-L1 and PD-L2 expression were higher in the low-risk group in all three patient cohorts, and PD-1 expression was higher in low-risk patients in the GSE10846 and GSE117556 cohorts. PD-1 and PD-L1 inhibitor therapy may therefore be more effective in low-risk group patients (Figure 8). We also examined the expression of several costimulatory (including *CTLA4*, *CD276*, *VSIR, IDO1, LAG3*, and *TIM-3*) and coinhibitory (including *GITR, CD27, CD40, ICOS, OX40*, *4-1BB*) checkpoint genes the GSE10846 (Figure 9), GSE31312 (Supplementary Figure 2), and GSE117556 (Supplementary Figure 3) cohorts. Drugs that targeting these checkpoints have been developed, but are either not yet widely used or still in early stages of the clinical trial process. We found that expression of most of these checkpoint genes differed significantly between the high-risk and low-risk groups. B7-CD28 gene family expression patterns might therefore aid in selecting ideal immunotherapies for individual patients in the future.

### B7-CD28-related immune signals in DLBCL

To determine the biological function of the B7-CD28 family in DLBCL, the 200 genes most strongly correlated with the B7-CD28 family (ranked by Pearson lRl) were identified in GSE10846 and GSE31312 cohort patients who were analyzed using the GPL570 platform (Figure 3). Gene ontology (GO) analysis (DAVID Bioinformatics Resource 6.8) was then performed to clarify the biofunctions of these genes (Supplementary Figure 4). Genes related to the B7-CD28 family were significantly enriched in immune response, T cell co-stimulation, adaptive immune response, and T cell receptor signaling pathway in patients from both cohorts. Owing to the close relationship between the B7-CD28 family and T cells and immunity [7, 17], we selected special biological process gene sets related to T cells from the AmiGO 2 Web portal. Gene set variation analysis (GSVA) [18] was then used to evaluate relationship between B7-CD28 genes, T cells, and immunity in the GSE10846 and GSE31312 datasets. B7- CD28 family gene expression was negatively correlated with T cell activation involved in immune response (GO:0002286), T-helper 1 type immune response (GO:0042088), and T-helper 2 type immune response (GO:0042092) (Supplementary Figure 5), indicating that the B7-CD28 family generally inhibits T cell immunity in DLBCL.

## DISCUSSION

In the past two decades, improved understanding of immune function in humans has led to the development of novel immune checkpoint inhibitors. Immunosuppressive agents and immune checkpoint inhibitors display considerable therapeutic effects against some tumors [19, 20]. DLBCL has been studied extensively in this context, but biomarkers that both predict clinical prognosis and immunotherapeutic responses while also reflecting the immune landscape in DLBCL tumors are lacking. Therefore, in this study, we established a prognostic model for DLBCL based on the B7-CD28 family and identified genes in this family that are significantly related to OS and PFS. Additionally, we investigated changes in the expression patterns of tumor-infiltrating lymphocytes to identify predictive immune markers that might serve as targets for immunotherapies targeting multiple immune checkpoints.

Diffuse large B-cell lymphoma research has focused extensively on immune checkpoints and the tumor microenvironment. Xu-Monette *et* *al*. showed that deficiency in NK, CD4+ T, and CD8+ T cell infiltration were associated with poor prognosis [21]. The following factors might help explain why we did not observe the same association in this study. First, while Xu-Monette *et* *al*. measured immune cell levels by examining several immunological markers, we estimated immune cell content using the powerful CIBERSORT algorithm, which estimates levels of 22 immune cells based on 547 genes. Our immune cell measurements might therefore be more accurate. Second, we subdivided immune cells into more categories. For example, we evaluated naive, memory resting, and memory activated CD4+ T cells, as well as resting and activated NK cells, separately in this study. Our results might therefore support more specific and detailed conclusions.

The core genes in our model were *CD80*, *CD86*, which belong to the B7 family, and *ICOS*, which belongs to the CD28 family. CD80 and CD86 are ligands for both the costimulatory receptor CD28 and the coinhibitory receptor CTLA-4; they are therefore crucial components of a major costimulatory pathway that regulates both T and B cell responses [22, 23]. CTLA-4 binds ligands with greater avidity and affinity than CD28 [7]. Unlike in many solid tumors, CD28 and CTLA-4 are innately expressed and play important biological roles in many hematological malignancies [24, 25]. For example, upregulation of CD80/CD86 and other costimulatory and adhesion molecules leads to increased APC activity and enhanced triggered T cell responses in follicular lymphoma (FL) [26]. CD80 and CD86 are also widely expressed in the hematological tumor microenvironment, and studies have shown that deletion of these genes may lead to failure of anti-tumor treatments [24, 27]. Here, we confirmed that elevated CD80 and CD86 expression were associated with a better prognosis. *ICOS* is one of the core genes of the B7-CD28 family; it is expressed primarily by activated T cells and binds to ICOS ligands (ICOSL) in APCs to regulate T helper cell 1 (Th1) and T helper cell 2 (Th2) activity [28, 29]. Some studies have shown that stimulating the ICOS pathway markedly enhances the efficacy of CTLA-4 blockade in cancer immunotherapy, while inhibiting the ICOS pathway reduces the efficacy of anti-CTLA-4 drugs and reduces tumor rejection [30, 31]. A recent study confirmed that regulatory T cells (Tregs) are produced through the ICOS/ICOSL pathway in FL, and that these ICOS^+^ Tregs inhibit the production of conventional T cells and FL B cells [32]. Additionally, Zhang *et al.* found that *ICOS* expression is negatively correlated with tumor metastasis, staging, and prognosis in colorectal cancer [33]. These results indicate that ICOS might be a valuable biomarker in various types of cancer.

The B7-CD28 family genetic model we describe in this study has some important limitations. Firstly, our data was obtained from the publicly-available GEO database; although it is comprehensive, this data is also retrospective, and prospective studies are needed to validate these results. Secondly, our model was based only on the B7-CD28 family of genes, and incorporation of additional gene expression data might improve the accuracy of its prognostic outcome predictions. Thirdly, although immunotherapy is a very promising treatment strategy, very few patients currently receive such treatments, and much additional research is needed. Finally, this study focused on molecular mechanisms in patients who were not treated with immune checkpoint inhibitors or agonists; the value of our model should therefore be confirmed in patients who did receive those treatments.

In conclusion, we established a prognostic model based on a B7-CD28 family three-gene signature, and the resulting risk score was significantly associated with OS and PFS. Based on this model, we identified an immune marker that predicted the distribution of some tumor-infiltrating lymphocytes and identified patients who benefited from immunotherapies targeting several immune checkpoints. These results not only help clarify the relationship between DLBCL and immune status, but may also help guide development of immunotherapies and individualized treatments for DLBCL patients.

## MATERIALS AND METHODS

### Patients and methods

Publicly available DLBCL patient clinical and gene expression data from the GSE10846 and GSE31312 datasets, which used the GPL570 platform, and the GSE117556 dataset, which used the GPL14951 platform, were obtained from the GEO database. After excluding patients for whom clinical data were not available, data from 1812 DLBCL patients were used in this study. 414 patients from the GSE10846 dataset were used as a training cohort, and 470 and 928 patients from the GSE31312 and GSE117556 datasets, respectively, were used as validation cohorts. The clinical characteristics of all patients are shown in Table 1. The following T cell-specific gene sets were downloaded from the AmiGO 2 Web portal (http://amigo.geneontology.org/amigo): GO:0002286: T cell activation involved in immune response; GO:0002424: T cell-mediated immune response to tumor cell; GO:0002840: regulation of T cell-mediated immune response to tumor cell; GO:0002842: positive regulation of T cell-mediated immune response to tumor cell; GO:0042088: T-helper 1 type immune response; and GO:0042092: T-helper 2 type immune response.

### Estimation of immune cell type fractions

The CIBERSORT algorithm estimates cell type proportions in a population based on bulk gene expression data. LM22 is a leukocyte gene signature containing 547 genes that are used to estimate human hematopoietic cell phenotypes, including B cells, T cells, NK cells, macrophages, dendritic cells, and myeloid subsets, with high accuracy. Using this algorithm, we estimated the fractions of twenty-two immune cell types in GSE10846 DLBCL samples and derived a P-value for each sample using Monte Carlo sampling at a threshold of *P* < 0.05. Then we compared the distribution of each immune cell in the established model using Student’s *t*-tests.

### Statistical analysis

Univariate Cox regression analysis was used to estimate the expression of each B7-CD28 family gene; genes with *P* < 0.05 were defined as survival-associated genes. The survival-related genes then were incorporated into a Cox proportional hazard regression model to establish a risk score equation. Risk scores were calculated for each patient; patients were then separated into high-risk and low-risk groups using the median risk score as the cutoff point. The Kaplan-Meier method was used to estimate and verify OS, and the log-rank test was used to compare survival differences between groups. Data were analyzed using SPSS version 21.0 and R software version 3.5.2, and a value of *P* < 0.05 was considered statistically significant.

## Supplementary Material

Supplementary Figures
