# Supplementary material for: B7-CD28 gene family expression is associated with prognostic and immunological characteristics of diffuse large B-cell lymphoma
Source: Aging (Albany NY). 2019 Jun 13;11(12):3939–57. doi: 10.18632/aging.102025 (PMC6629000; doi:10.18632/aging.102025)
Supplement: Supplementary Figures [file aging-11-102025-s001.pdf]

SUPPLEMENTARY FIGURES

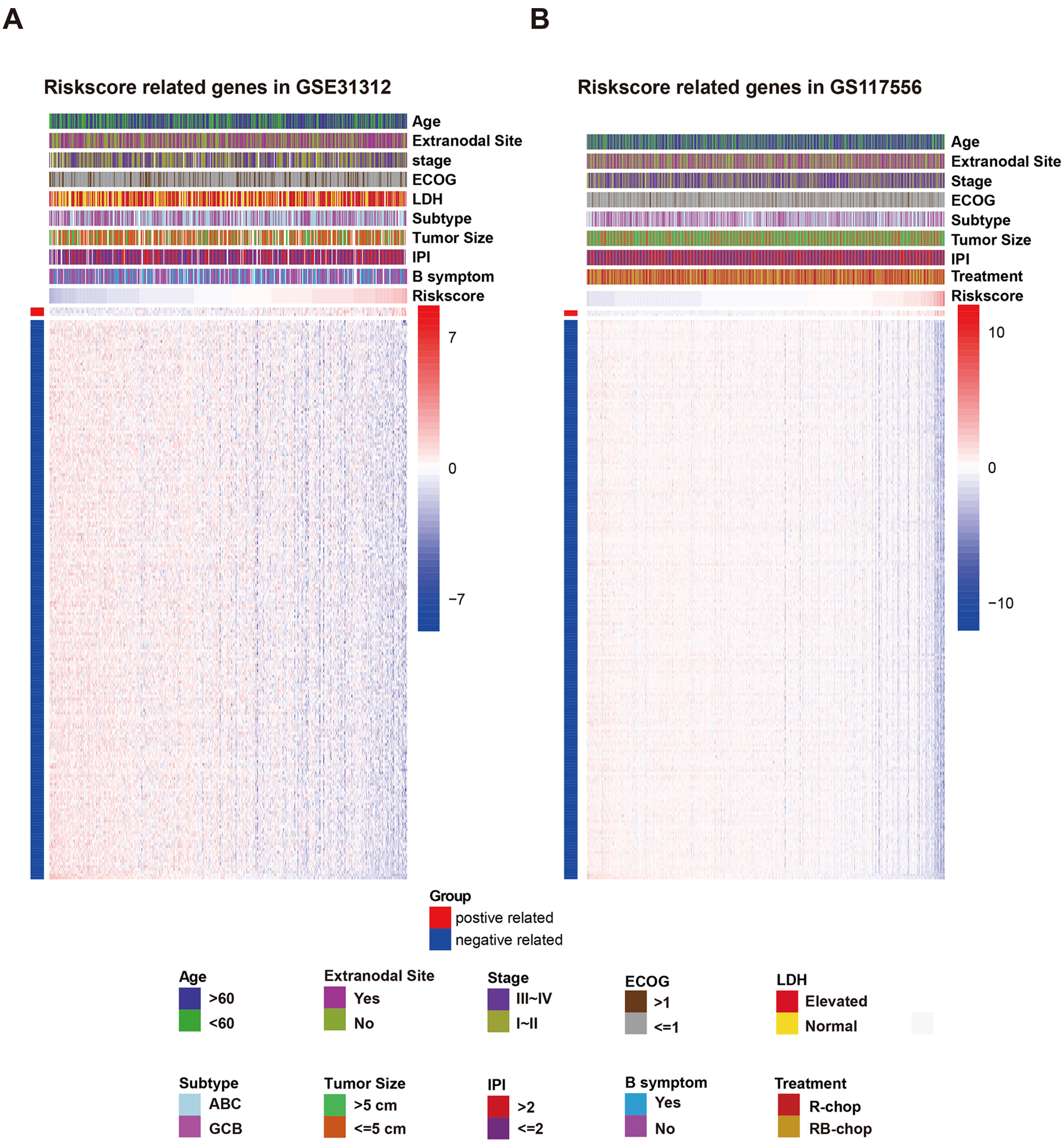

Supplementary Figure 1. Risk score-related genes and clinical characteristics in GSE31312 (**A**) and GSE117556 (**B**) patients. ECOG: Eastern Cooperative Oncology Group score, IPI: International Prognostic Index, ABC: activated B-cell-like, GCB: germinal center B-cell-like, R-CHOP: cyclophosphamide, doxorubicin, vincristine, and prednisone plus rituximab, BR-CHOP: cyclophosphamide, doxorubicin, vincristine, and prednisone plus rituximab and bortezomib.

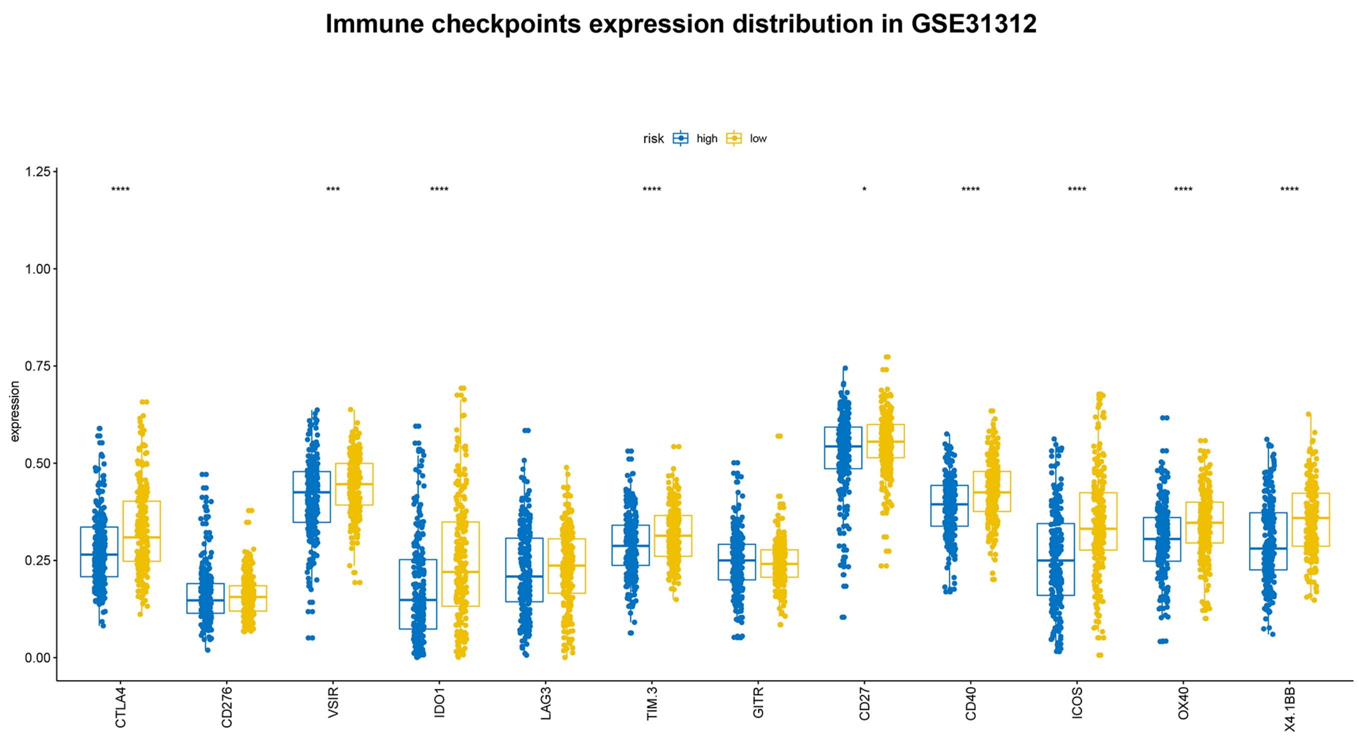

Supplementary Figure 2. Distribution of immune checkpoint gene expression in GSE31312 patients.

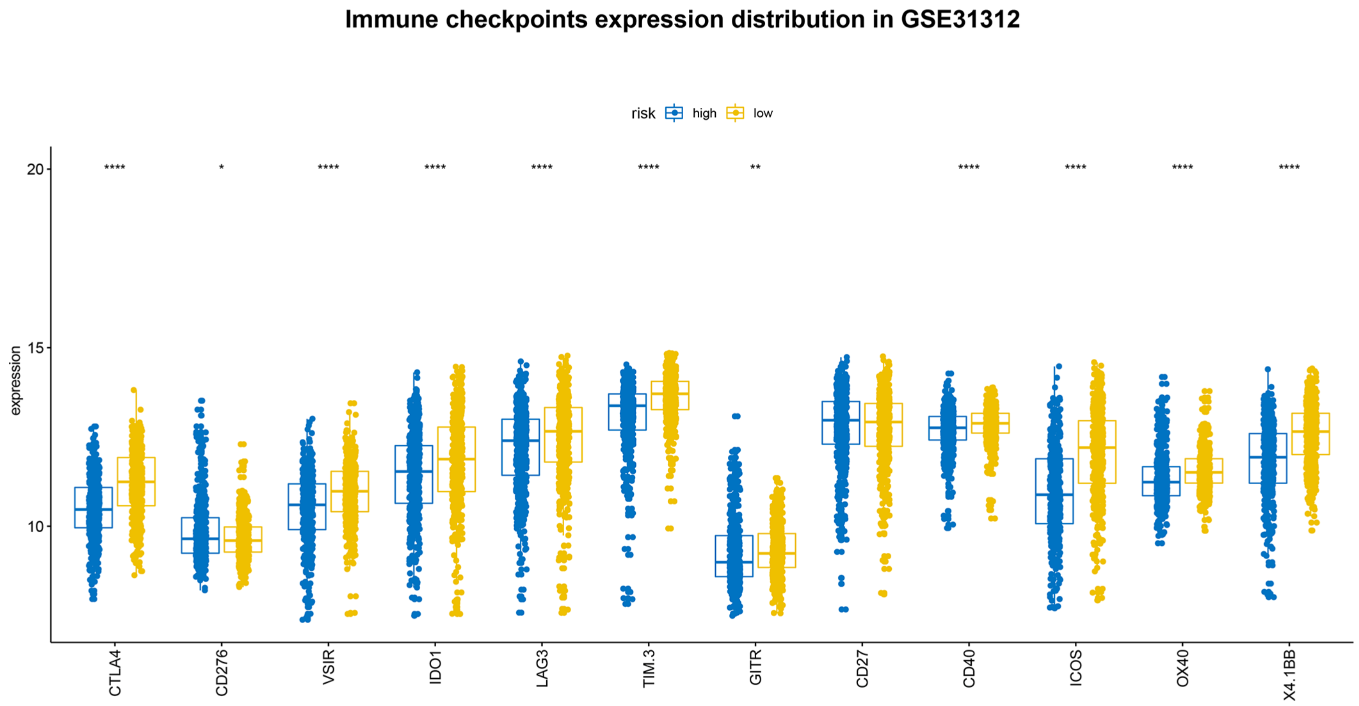

Supplementary Figure 3. Distribution of immune checkpoint gene expression in GSE117556 patients.

A

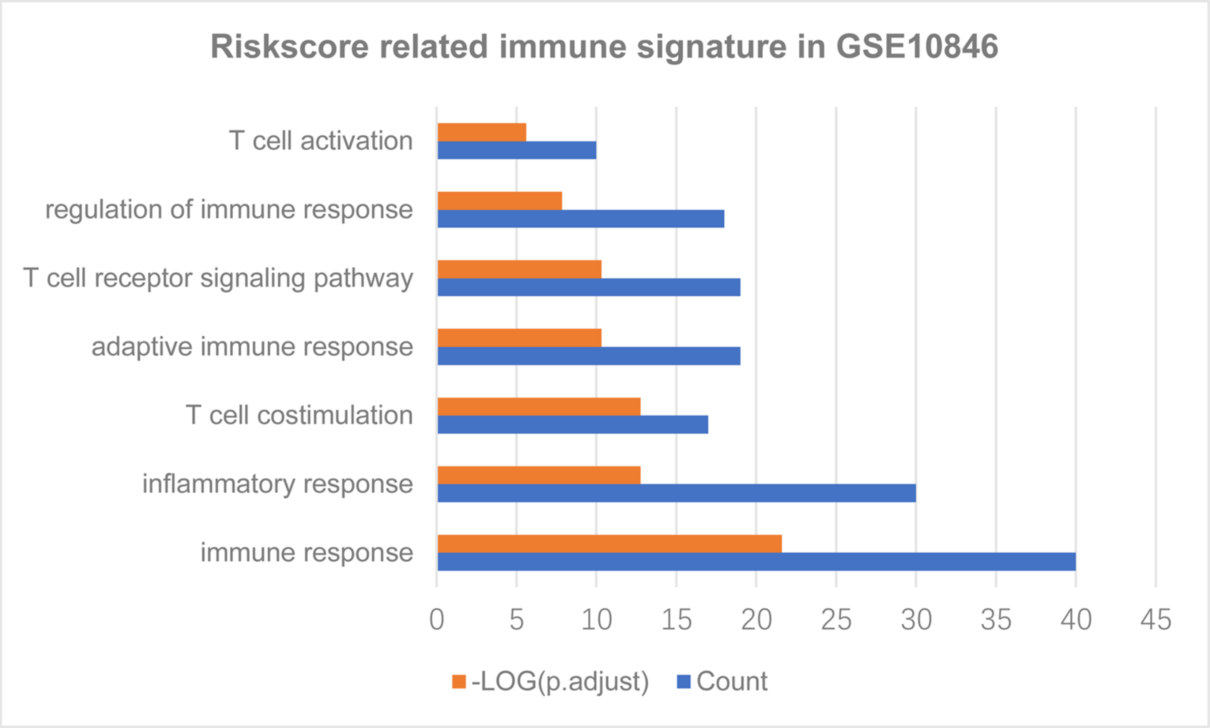

B

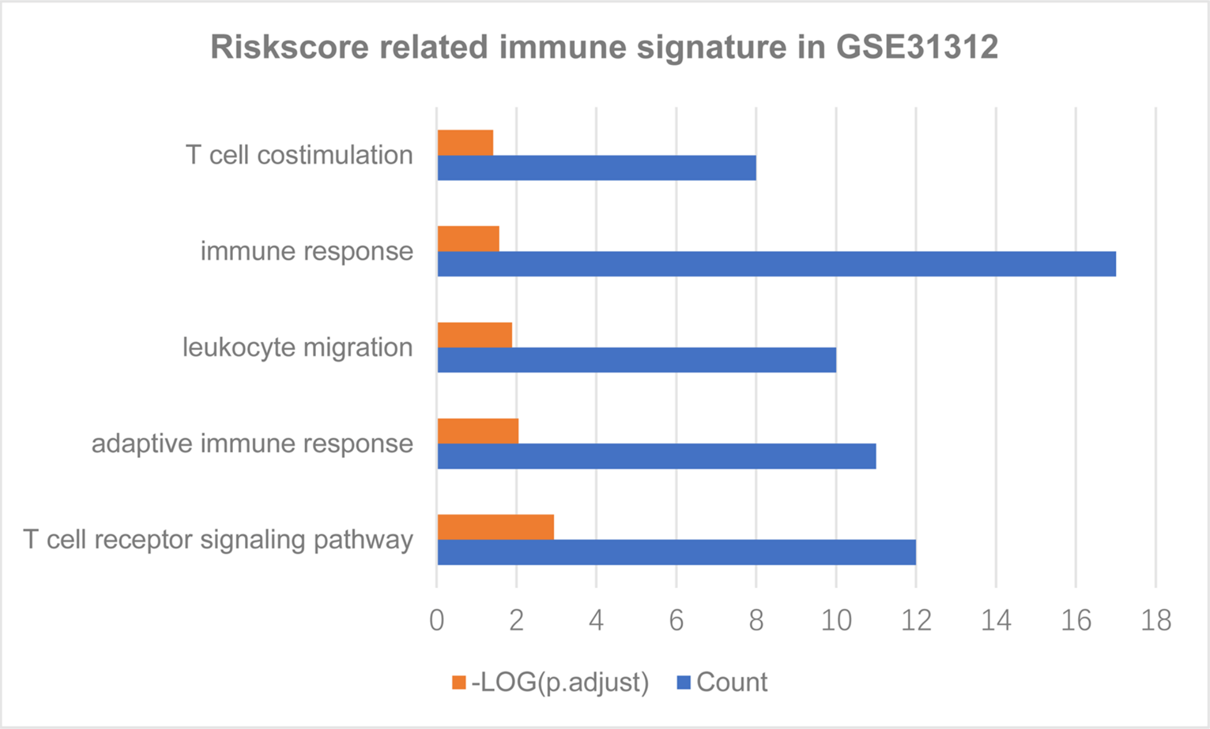

Supplementary Figure 4. Risk score-related immune expression patterns in GSE10846 (**A**) and GSE31312 (**B**) patients.

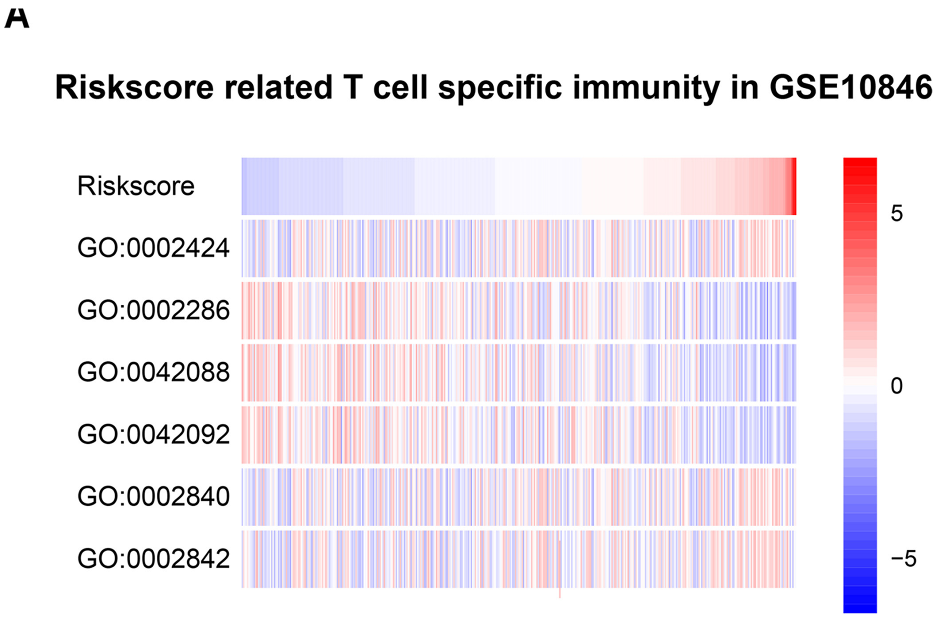

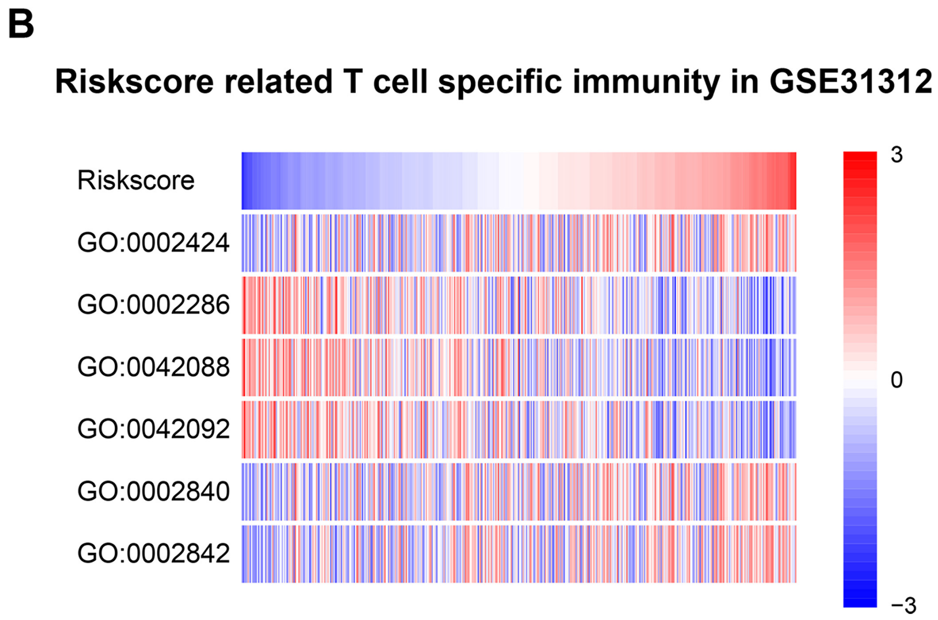

Supplementary Figure 5. Risk score-related T cell-specific immunity in GSE10846 (**A**) and GSE31312 (**B**) patients.
